# Developmental patterns of intestinal group 3 innate lymphoid cells in piglets and their response to enterotoxigenic *Escherichia coli* infection

**DOI:** 10.1186/s13567-024-01418-3

**Published:** 2024-12-18

**Authors:** Ningning Huang, Ling Ye, Hao Li, Jian Peng, Hongkui Wei

**Affiliations:** 1 Department of Animal Nutrition and Feed Science, College of Animal Science and Technology, Huazhong Agricultural University, Wuhan, 430070 China; 2 Center of Cellular and Genetic Sciences, Henan Academy of Sciences, Zhengzhou, 450000 China; 3 Cooperative Innovation Center for Sustainable Pig Production, Wuhan, China

**Keywords:** ILC3s, NKp46^+^ILC3, NKp46^−^ILC3, suckling piglets, enterotoxigenic *Escherichia coli*

## Abstract

Diarrhoea and preweaning mortality in piglets are crucial factors impacting the economic sustainability of the swine industry. Pathogenic infections are among the main causes of diarrhea and mortality. Group 3 innate lymphoid cells (ILC3s) are crucial for safeguarding against pathogenic infections. However, knowledge regarding the development and function of ILC3s in suckling piglets is currently limited. Our findings demonstrate that the development of ILC3s in suckling piglets gradually progresses from day 1 to day 21, with a notable increase observed on day 28. Additionally, the development of NKp46^+^ILC3s and the production of interleukin (IL)-17A by ILC3s displayed consistent patterns with the changes observed in ILC3s. Notably, interferon (IFN)-γ levels significantly increased on day 14. Moreover, the production of IFN-γ by NKp46^+^ILC3s was greater than that by NKp46^−^ILC3s. Importantly, when piglets were subjected to a 4-h challenge with enterotoxigenic *Escherichia coli*, both the percentages of ILC3s significantly increased, accompanied by increased IL-22 production, highlighting their importance in maintaining intestinal health. The outcomes of this study provide valuable insights for future related research.

**Supplementary Information:**

The online version contains supplementary material available at 10.1186/s13567-024-01418-3.

## Introduction

Piglet mortality has long posed a significant challenge to the pig industry, with a reported preweaning mortality rate of 10% [1]. The intestinal immune system of piglets is immature at birth and undergoes maturation at approximately 7 weeks of age [2]. During the early stages of life, neonatal pigs are highly susceptible to pathogens, which can lead to diarrhea and even death [3]. Research has shown that enterotoxigenic *Escherichia coli* (ETEC) is one of the main pathogens responsible for causing diarrhoea in suckling piglets [3, 4].

The porcine intestine not only acts as the site for the digestion and absorption of nutrients but is also the largest immune organ. Intestinal immunity, in a state of homeostasis, restricts the access of potentially harmful organisms and enteropathogenic microorganisms to the intestine, thus preserving intestinal health. In humans and mice, group 3 innate lymphoid cells (ILC3s) constitute the most abundant subset of innate lymphoid cells (ILCs) [5]. ILC3s were originally found in foetuses, and development after birth depends on retinoid orphan receptor gamma t (RORγt) [6]. During early life, ILC3s are the main source of interleukin (IL)−17A and IL-22, which are important for the migration of neutrophils during infection and for barrier tissue regeneration and maintenance, respectively, in the immature adaptive immune system [7]. ILC3s encompass heterogeneous populations in mice, consisting of the natural cytotoxicity receptor NKp46^+^ILC3, CCR6^+^ILC3, and CCR6^−^NKp46^−^ILC3 subsets [8–10]. At birth, the majority of RORγt^+^ILC3s are CCR6^+^ILC3s, which exhibit numerous similarities with lymphoid tissue inducer (LTi) cells and can secrete IL-17A and IL-22 [9]. Then, NKp46^+^ILC3s undergo rapid expansion until 4 weeks after birth and exhibit increased production of interferon (IFN)-γ [9, 11], which is required for *Salmonella* typhimurium resistance [12]. From a functional perspective, ILC3s play a crucial role in maintaining intestinal epithelial integrity through the production of these cytokines in response to pathogen infections [12–14].

However, the understanding of porcine ILC3s remained limited until the expression of ILCs markers during gestation in pigs [15]. In previous studies, porcine ileal ILC3s were identified and characterized by single-cell RNA sequencing [16]. ILC3s are the most abundant ILCs in the lamina propria lymphocytes (LPLs) of the jejunum [17]. Importantly, porcine ILC3s express many surface receptors, including CD2, KIT, IL-7Rα, IL-23R, natural cytotoxicity receptors (NCRs), and aryl hydrocarbon receptors (AHRs), and it has been reported that human and pig ILCs share more gene signatures than do mouse ILCs. ILC3s can be divided into different subsets according to their gene expression profile [17]. Consequently, subsets of ILC3s can be identified via flow cytometry by considering the single-cell gene expression profiles reported in previous studies [16, 17].

In human and mouse studies, ILC3s have been reported to play a role in the defense against extracellular pathogens, epithelial tissue homeostasis, and the modulation of inflammation by producing cytokines [18]. However, the development of intestinal ILC3s and the pattern of ILC3 cytokine production in piglets during the suckling period are not known. To address this knowledge gap, this study first measured the levels of IL-17A, IL-22, and IFN-γ in the plasma and intestine of pigs throughout the suckling period. The development of ILC3s and ILC3 subsets in the jejunum of piglets before weaning was subsequently revealed via flow cytometry, and the production and sources of those cytokines during homeostasis were further examined. These results further indicate that ILC3-derived IL-22 is an important immune response to ETEC infection. In summary, this study provides insights into the development and function of ILC3s in piglets before weaning, providing a reference for intestinal health research.

## Materials and methods

### Animals and sampling

First, sows that were free from clinical disease on a pig farm located in Wuhan, Hubei Province, were selected for this study. Suckling crossbred piglets (Duroc–Landrace–Yorkshire) from different litters (one piglet per litter) in the same building with similar weights, no access to probiotics or antibiotics, and no diarrhea were selected to study their cellular development at 1-day-old, 7-day-old, 14-day-old, 21-day-old and 28-day-old piglets (preweaning) (*n* = 4–5), respectively. A total of 21 piglets were purchased from a pig farm located in Wuhan, Hubei Province. During the entire experimental period, the nutrient requirements of lactating sows were met as recommended by the National Research Council [19]. The biological sex of the pigs was chosen randomly. All the piglets had free access to sow milk and water during the suckling period. For sample collection, suckling piglets were euthanized, and plasma was collected and stored after centrifugation at 3000 rpm for 10 min. Jejunal samples were immediately frozen in liquid nitrogen and stored at −80 °C for cytokine assays.

### ETEC challenge

ETEC (CVCC225, O8: K87: K88ac) were cultured in nutrient broth and incubated at 37 °C with shaking for 12–16 h or overnight. ETEC were harvested via centrifugation at 5000 rpm for 10 min, washed three times with sterile phosphate-buffered saline (PBS), resuspended in sterile PBS, and diluted to 2 × 10^9^ CFU/mL for piglet challenge. A total of 16 crossbred Duroc × Landrace × Yorkshire piglets were reared by sows and weaned at 21 days of age. Piglets (16 piglets from eight different litters) were individually housed in metabolic cages and randomly assigned to four groups, with four piglets in each group (two males and two females). After adapting to their surroundings for 3 days, all the piglets were given 10 mg/mL streptomycin to disrupt their normal intestinal microbiota. After 24 h, all the piglets were challenged with 50 mL of nutrient broth containing 2 × 10^9^ CFU/mL ETEC. The diarrhea state of the piglets was observed.

### Cytokine assays in the plasma and jejunum

The levels of IL-17A, IL-22, and IFN-γ in the plasma and jejunum were evaluated via the Porcine IL-17A ELISA Kit (2P-KMLJ941949p), Porcine IL-22 ELISA Kit (2P-KMLJ941955p), and Porcine IFN-γ ELISA Kit (2P-KMLJ941940p) purchased from Nanjing Camilo Biological Engineering Co., Ltd., as per the manufacturer’s protocol. In all the assays, the mean OD_600nm_ reads obtained from duplicate wells of the diluent negative controls were subtracted from all the readings (samples and controls) before standard curves were constructed, from which the sample concentrations were calculated. The concentrations are expressed as pg/mL or pg/g.

### Isolation of intestinal LPLs

For the isolation of the jejunum, approximately 5 cm of the jejunum from each piglet was collected and stored in PBS on ice. The outer muscle layer and serosa were peeled off, and the intestine was cut longitudinally. The tissue was rinsed gently with PBS to remove the intestinal contents and cut into 1-cm fragments with scissors. These fragments were shaken and digested in separation solution containing 20 mL of Hank’s balanced salt solution (HBSS), 1% penicillin and streptomycin, 1% 4-(2-hydroxyethyl)−1-piperazineëthanesulfonic acid (HEPES), 5 mM ethylenediaminetetraacetic acid (EDTA) and 0.94 mol/L dithiothreitol (DTT) and incubated in a shaking incubator at 37 °C and 200 rpm for 40 min. The intestinal fragments were sieved to remove the liquid, placed in an Erlenmeyer flask, and 20 mL of prewarmed separation solution was added. This procedure was repeated three times (40 min each) to digest the intestinal epithelium. After shaking gently for approximately 2 min with 20 mL of HBSS without Ca/Mg, the fragments were transferred to a solution containing 20 mL of RPMI-1640 medium, 1% penicillin and streptomycin, 2 mM HEPES, 2 mg of DNase I, and 2% fetal bovine serum (FCS) and incubated in a shaking incubator at 37 °C and 200 rpm for 30 min to restore ionic homeostasis. Fragments were collected after shaking, placed into digestive juice solution (10 mL of RPMI-1640 medium, 1% penicillin and streptomycin, 2 mM HEPES, 10 mg of DNase I, 50 mg of collagenase VIII and 1% heat-inactivated FCS) and incubated for 60 min in a shaking incubator (200 rpm) at 37 °C. The intestinal fragments were sieved through a 70 μm cell strainer, and the mixture was collected. Culture solution (10 mL of RPMI-1640 and 5% FCS) was added to the collected digestive juice, and the mixture was centrifuged at 4 °C and 400 × *g* for 10 min, resuspended in 40% Percoll solution (GE Healthcare), slowly added to 80% Percoll solution, and centrifuged at 24 °C and 800 × *g* for 20 min. Mononuclear cells were then harvested from the interphase of 40% and 80% Percoll, washed three times, and resuspended in sterile PBS with 1% FCS. The method used for the isolation of colonic leucocytes was similar to that used for the jejunum.

### Flow cytometry

The monoclonal antibodies used in the experiments were as follows: Fixable Viability Stain 510 (BD Pharmingen), IFN-γ (IC985G, R&D Systems), CD3ε (BB23-8E6-8C8) (561477, BD Pharmingen), CD3ε (BB23-8E6-8C8) (559582, BD Pharmingen), CD127 (17–1278-42, Thermo), CD127 (HIL-7R-M21) (560822, BD Pharmingen), IL-22 (11–7229, Thermo), CD45 (HI30) (563879, BD Pharmingen), CD45 (K252.1E4) (MCA1222A647, Bio-Rad), IL-17A (11–7179, Thermo), CD335 (NKp46) (MA528352Thermo), γδT (MAC320) (561482, BD Pharmingen), CD8α (76–2–11) (559584, BD Pharmingen), CD2 (12–0029, Thermo), CD19 (557791, BD Pharmingen), CD14 (555398, BD Pharmingen), and RORγt (IC6006C, RD Systems). Cells with the phenotypes of ILC3s (CD45^+^CD3^−^CD14^−^CD19^−^CD127^+^RORγt^+^), NK cells (CD3^−^CD8α^+^CD2^+^), T cells (CD45^+^CD3^+^) and γδT cells (CD45^+^CD3^+^γδT^+^) were identified using flow cytometry. The percentages of cells were measured using the Beckman Coulter Cytoflex-LX flow cytometer (Beckman Coulter, CA, USA). The proportion of ILC3s or subset populations among total viable single cells can be calculated by dividing the cell count for a particular ILC3 subset by the number of live single cells. The acquired data were analysed via FlowJo-V10.8.1 software.

For surface marker staining, approximately 5 × 10^6^ cells were placed in 1.5 mL Eppendorf (EP) tubes, Fc blockers were added at a ratio of 1:100, mixed evenly, and incubated at room temperature for 5 min in the dark. Fixable viability stain and mixed antibodies were added, and the samples were incubated at 4 °C for 30 min in the dark. For nuclear staining, the cells were fixed and permeabilized using Transcription Factor Buffer Set working solution according to the manufacturer’s instructions and incubated at 4 °C for 45 min. PBS buffer was added, and the mixture was centrifuged at room temperature (3000 r/min) for 5 min to dilute the transcription factor buffer. For detection of intracellular cytokine production, the cells were stimulated with phorbol-12-myristate13-acetate (PMA) (50 ng/mL) and ionomycin (500 ng/mL, Sigma‒Aldrich) for 2 h and brefeldin (2 μg/mL, BioLegend) for 2 h. IL-17A, IL-22, and IFN-γ were added for staining and incubated at 4 °C for 30 min before the cells were harvested for analysis. The cells were sifted with a cell strainer (70 μm), and the data were detected and collected. The data were analysed via FlowJo-V10.8.1 software.

### Statistical analysis

Normality, lognormality, and Shapiro‒Wilk tests were performed for normality tests before data analysis, with the significance level set at 0.05. Unpaired one-way analysis of variance or the Kruskal–Wallis test was used for analysis according to whether the data were normally distributed, and Dunn’s multiple comparison test was used for post hoc tests. Correlation analysis was performed via GraphPad Prism 8.3.0 software, and the data are presented as the means ± standard errors. *P* < 0.05 indicated a significant difference, and *P* < 0.01 indicated a very significant difference.

## Results

### Plasma and jejunal levels of IL-17A, IL-22, and IFN-γ

**Figure 1:**
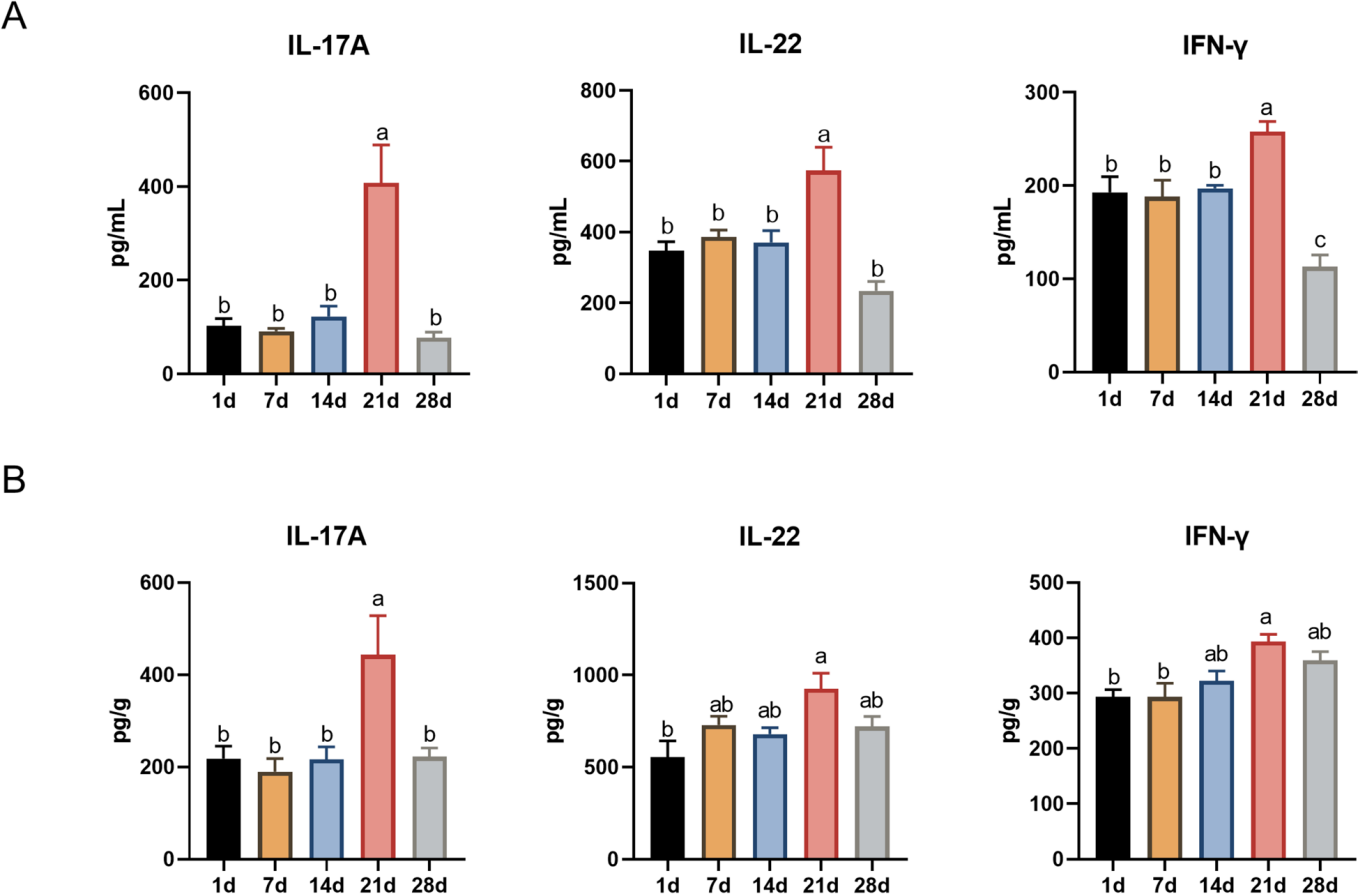
**Plasma and jejunal levels of IL-17A, IL-22, and IFN-γ.** The levels of IL-17A, IL-22, and IFN-γ in the plasma were determined by ELISA (**A**). Cytokine levels in the jejunum of piglets (**B**). The data are presented as the means ± standard errors, *n* = 4–5. ^a−c^Different lowercase letters represent significant differences (*P* < 0.05).

IL-17A, IL-22, and IFN-γ are tissue-signalling cytokines that play important roles in inflammation and infection [20, 21]. This study determined the levels of cytokines in the plasma and jejunum of suckling piglets. There were no significant differences in the levels of IL-17A, IL-22, or IFN-γ between days 7 and 14 and day 1. Although their levels significantly increased at 21 days after birth (*P* < 0.05), the levels of cytokines on day 28 returned to the same levels as those at days 1 to 14 (*P* < 0.05) (Figure 1A). A similar trend was observed in the levels of IL-17A, IL-22, and IFN-γ in the jejunal tissues of the piglets (Figure 1B). These results suggest that as the piglets aged, the levels of IL-17A, IL-22, and IFN-γ in both plasma and jejunal tissues gradually increased early, followed by a rapid surge on day 21 after birth and a subsequent decline before weaning.

### Potential cells produce IL-17A, IL-22, and IFN-γ in the jejunal lamina propria of piglets

**Figure 2:**
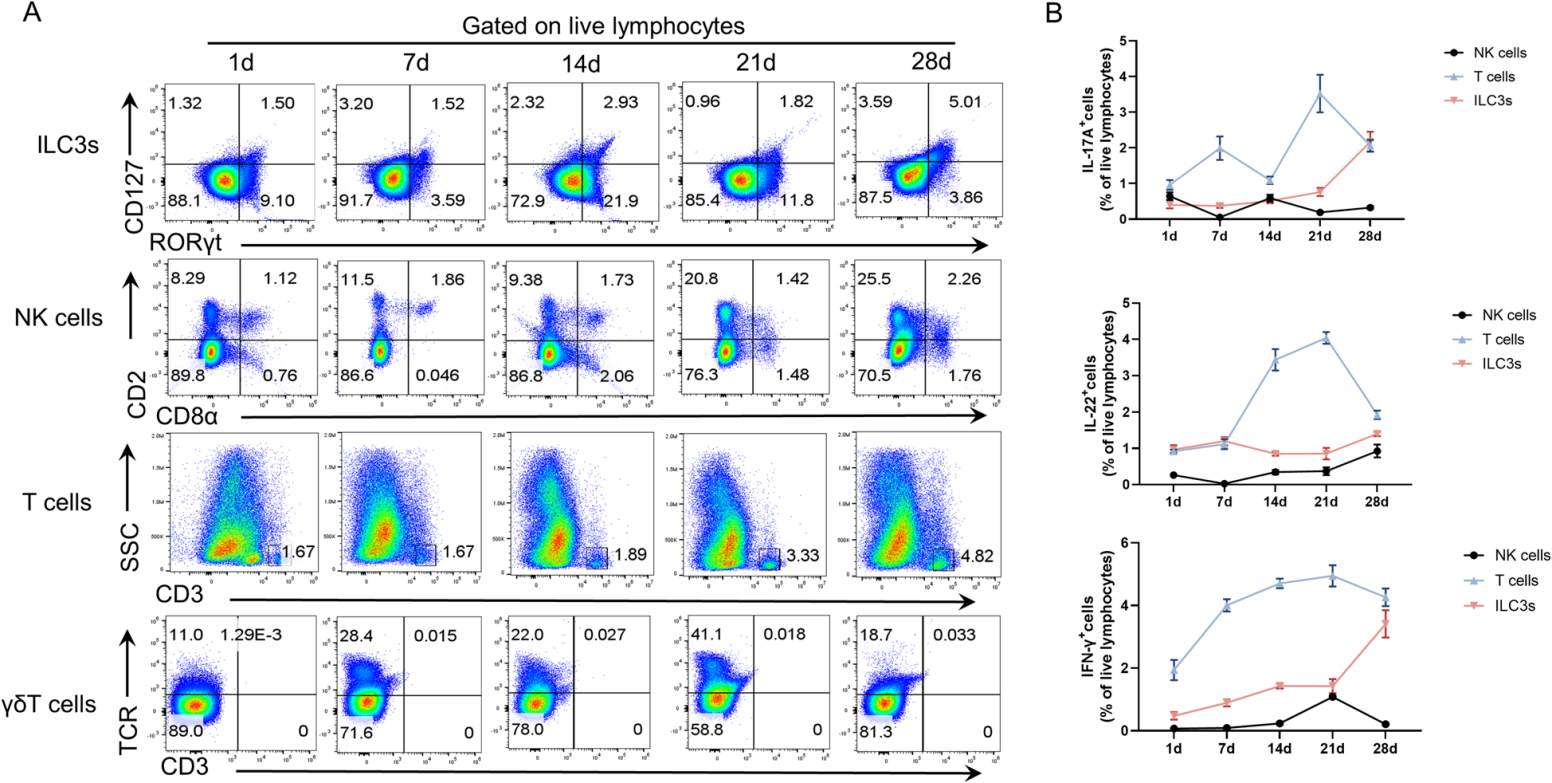
**Potential cells producing IL-17A, IL-22, and IFN-γ in the jejunal lamina propria of piglets.** Jejunal lamina propria lymphocytes (LPLs) were isolated from 1- to 28-day-old piglets (**A, B**). The proportions of ILC3s, NK cells, γδT cells and T cells gated on live lymphocytes. The numbers in the flow plots represent the percentages of cells on each gate (**A**). The expression of IL-17A, IL-22, and IFN-γ in ILC3s, NK cells, and T cells (**B**). The data are presented as the means ± standard errors (*n* = 4–5).

Human and mouse studies have shown that ILC3s, natural killer (NK) cells, γδT cells, and T cells are the main intestinal immune cells that produce IL-17A, IL-22 and IFN-γ [22–31]. Therefore, this study further examined the proportions of these cells in the jejunal LPLs of suckling piglets. The analysis revealed that the proportions of NK cells and γδT cells among live lymphocytes remained consistent throughout the entire observation period. Conversely, the percentages of ILC3s and T cells did not significantly change from days 1 through 21 but notably increased on day 28 after birth (Figure 2A). Importantly, the proportion of ILC3s gated on live lymphocytes was slightly greater than that of NK cells and γδT cells during the whole period. Furthermore, the proportion of T cells was similar to that of ILC3s (Figure 2A). This study further analysed the expression of these cytokines in these cell types and revealed that T cells presented increased expression levels of IL-17A, IL-22, and IFN-γ. However, the expression of cytokines was negligible in γδT cells. In contrast to those produced by NK cells, the ILC3s that produced these cytokines presented slightly elevated levels (Figure 2B). These results indicate that T cells primarily contribute to the adaptive immune response by producing IL-17A, IL-22, and IFN-γ, whereas ILC3s serve as the main innate source of these cytokines.

### Development and distribution of intestinal ILC3s in piglets

**Figure 3:**
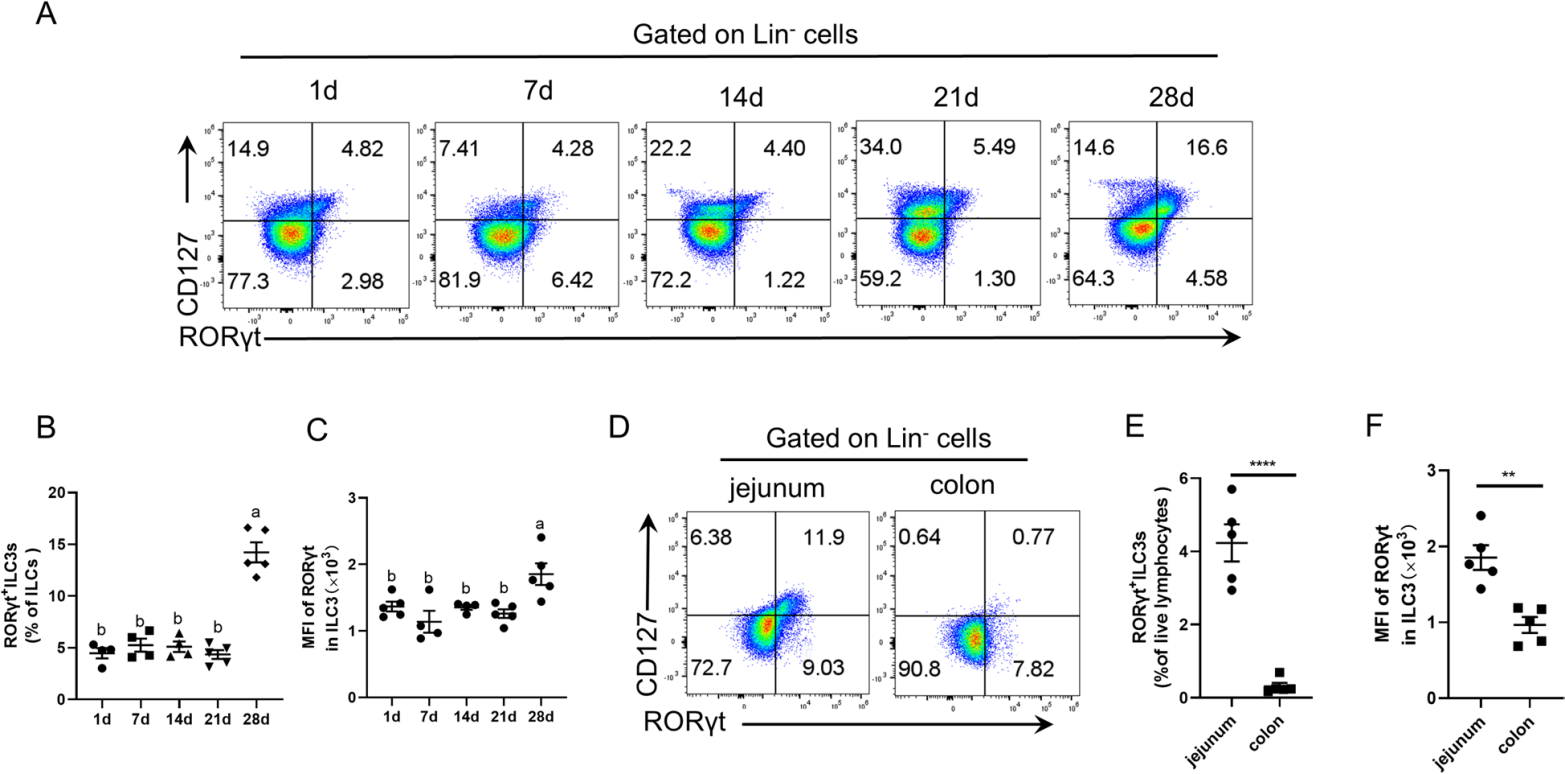
**Development and distribution of intestinal ILC3s in piglets.** Flow cytometric analysis of ILC3s in lineage-negative cells isolated from the jejunal and/or colon lamina propria of piglets. The numbers in the flow plots represent the percentages of RORγt^+^ILC3s at the lineage gates (**A**). Percentages of ILC3s in total ILCs (**B**). The mean fluorescence intensity (MFI) of RORγt in RORγt^+^ILC3s (**C**). Plots were gated on lineage-negative cells in jejunal and colon LPLs of 28-day-old piglets (**D**). Percentages of ILC3s in total live lymphocytes (**E**) and the MFI of RORγt in RORγt^+^ILC3s (**F**). The data are presented as the means ± standard errors, *n* = 4–5. ^a–b^Different lowercase letters represent significant differences (*P* < 0.05). *P < 0.05, **P < 0.01, ***P < 0.001, ****P < 0.0001.

ILC3s are tissue-resident cells that are enriched in the intestine [8]. To explore the development of intestinal ILC3s in piglets, this work isolated and analysed jejunal LPLs from suckling piglets at different ages. To identify ILC3s, CD127- and RORγt-copositive cells were selected, and a gating strategy was used (Additional file 1A). The results revealed that the proportions of ILC3s among lineage-negative cells or total ILCs and the RORγt mean fluorescence intensity (MFI) of RORγt^+^ILC3s remained constant from days 1 through 21 but significantly increased on day 28 (*P* < 0.05) (Figures 3A–C). A growing body of literature shows that ILC3s are widely distributed in the body [32]. The present study further detected the distribution of ILC3s in the jejunum and colon of pigs, revealing that the proportions of RORγt^+^ILC3s and the RORγt MFI of RORγt^+^ILC3s in the jejunum were significantly greater than those in the colon on day 28 (Figures 3D–F). Therefore, these findings demonstrated that the percentages of RORγt^+^ILC3s and the MFI of RORγt in RORγt^+^ILC3s were low early in suckling pig development and increased rapidly on day 28 in the jejunum and that ILC3s were predominantly enriched in the small intestine.

### Cytokines from jejunal ILC3s in suckling piglets

**Figure 4:**
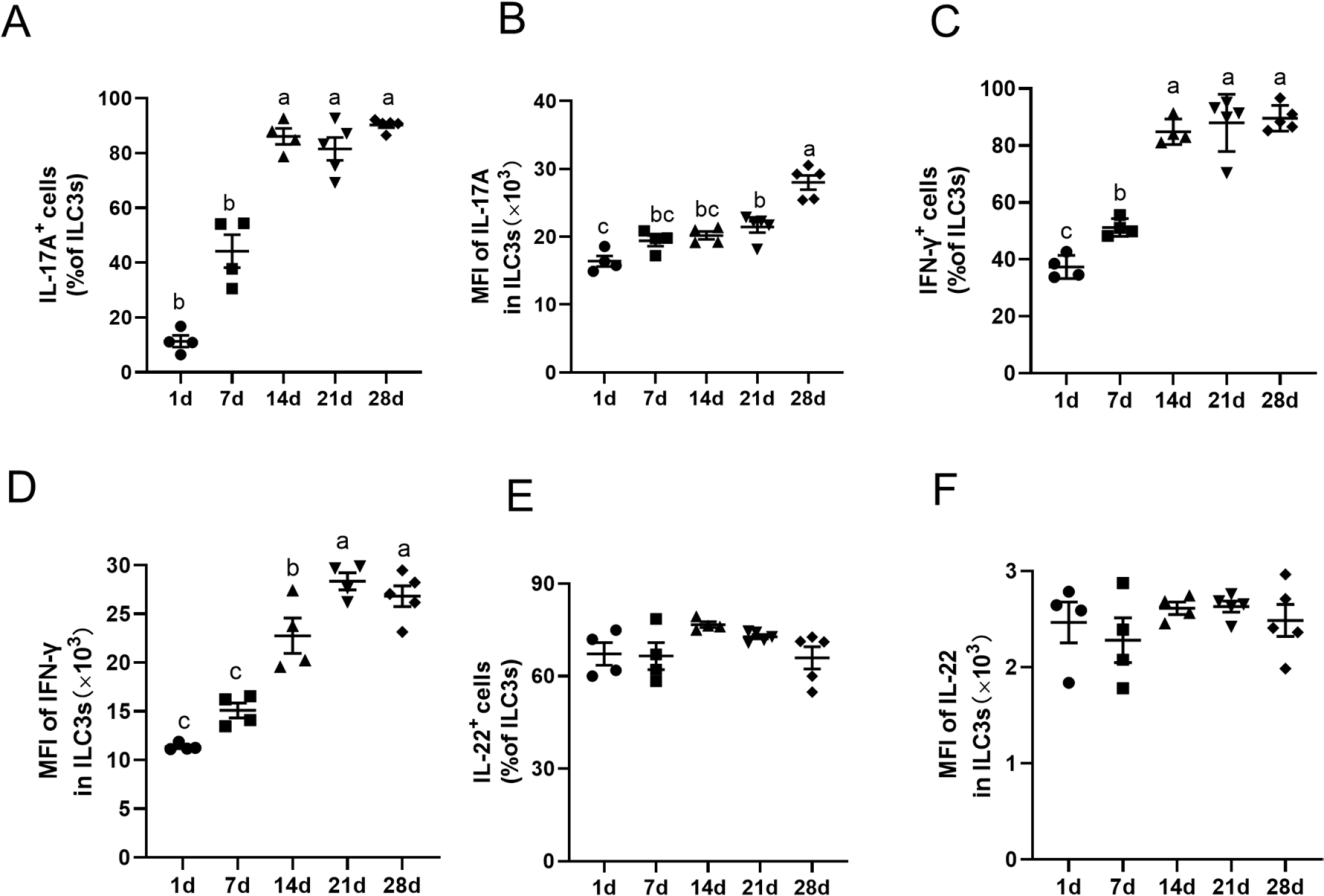
**Cytokines from jejunal ILC3s of suckling piglets.** The percentages of IL-17A production in jejunal ILC3s were analysed by flow cytometry (**A**). The MFI of IL-17A in ILC3s (**B**). The percentages of IFN-γ-producing cells in jejunal ILC3s (**C**). The MFI of IFN-γ in ILC3s is shown (**D**). Percentages of IL-22-producing ILC3s (**E**). The MFI of IL-22 in ILC3s (**F**). The data are presented as the means ± standard errors, *n* = 4–5. ^a–b^ Different lowercase letters indicate significant differences (*P* < 0.05).

ILC3s are important innate sources of IL-17A, IL-22 and IFN-γ, which play crucial roles in regulating intestinal infection and maintaining homeostasis [14, 33]. A gating strategy was used to identify these cytokines from ILC3s via flow cytometry (Additional file 1B). To further reveal the function of jejunal ILC3s, this study analysed the percentages of cytokine-producing ILC3s, as well as the MFI values of these cytokines. The percentages of IL-17A in ILC3s markedly increased at days 14 through 28 (Figure 4A). Additionally, the IL-17A MFI of IL-17A^+^ ILC3s was also increased on day 28 in the jejunum of suckling piglets (Figure 4B). Furthermore, a significant increase in the frequency of IFN-γ^+^ILC3s, as well as the MFI of IFN-γ in ILC3s, was observed on day 14 in the jejunum, after which the frequency of IFN-γ^+^ILC3s remained stable (Figures 4C and D). In contrast, the level of IL-22 derived from ILC3s did not significantly change during the whole period (Figures 4E and F). Collectively, these findings indicate that as the age of piglets increases, developmental changes in the production of IL-17A and IFN-γ by ILC3s occur.

### Development of jejunal ILC3 subsets in suckling piglets

**Figure 5:**
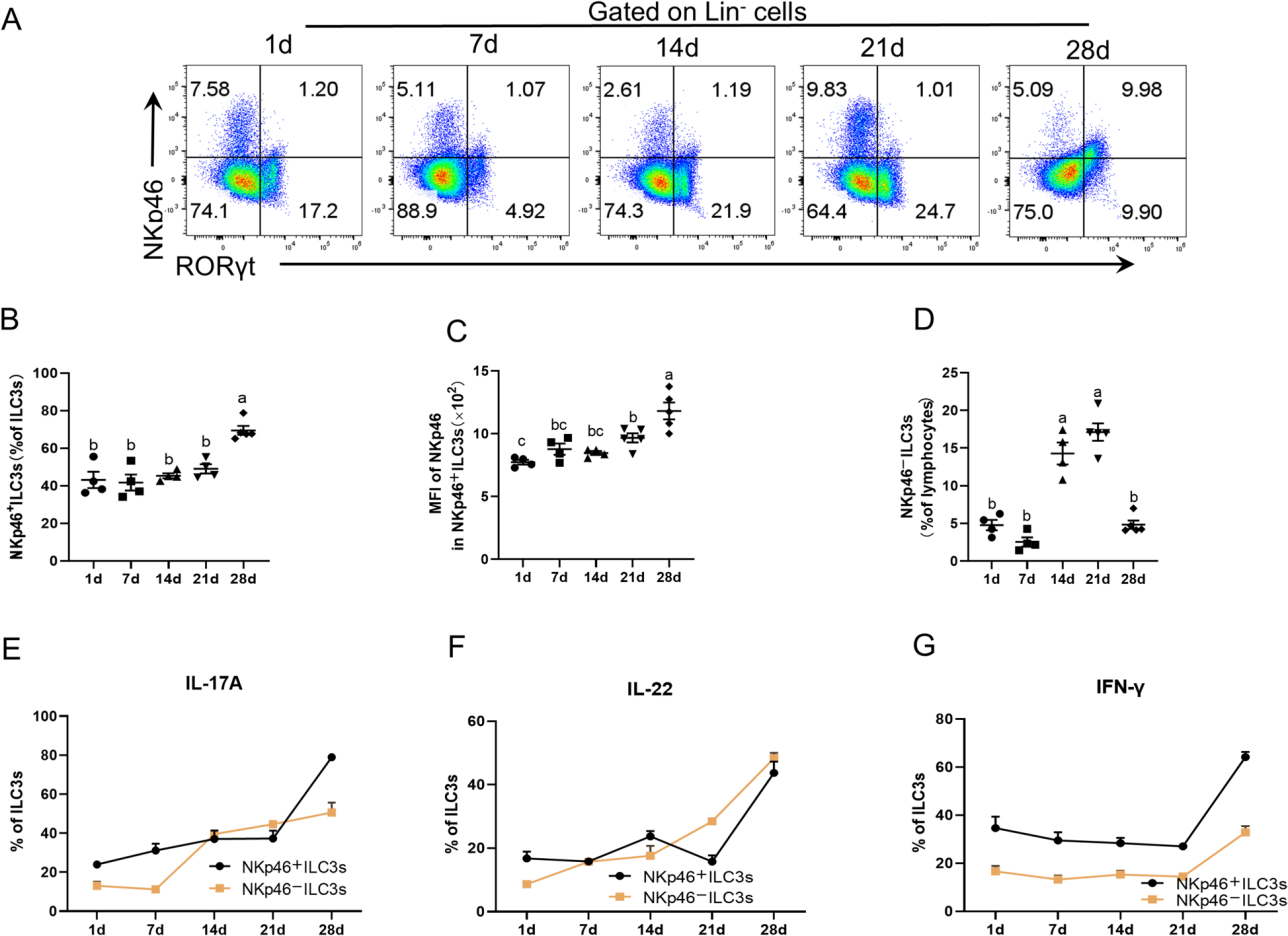
**Development of jejunal ILC3 subsets in suckling piglets. **Jejunal lamina propria lymphocytes (LPLs) were isolated from 1 to 28-day-old piglets. NKp46^+^ILC3s and NKp46^−^ILC3s were analysed by flow cytometry (A–G).The expression of NKp46 and RORγt in lineage negative cells (**A**). Percentages of NKp46^+^ILC3s in ILC3s (**B**). MFI of NKp46 in NKp46^+^ILC3s (**C**). Percentages of NKp46^−^ILC3s gated on live lymphocytes (**D**). percentages of IL-17A (**E**), IL-22 (**F**), and IFN-γ (**G**) production in NKp46^+^ILC3s and/or NKp46^−^ILC3s. The data are presented as the means ± standard errors, *n* = 4–5. ^a–b^Different lowercase letters represent significant differences (*P* < 0.05).

In mice, ILC3s can be divided into two subsets on the basis of the presence or absence of NKp46, and these subsets exhibit distinct cytokine expression profiles [9]. In addition, NKp46^ −^ILC3s are considered potential precursors of NKp46^+^ILC3s [11, 34]. To further explore the development of NKp46^+^ILC3s and NKp46^−^ILC3s from suckling piglets, this study measured the percentages of NKp46^+^ILC3s and NKp46^−^ILC3s among lineage-negative cells (Figure 5A), total ILC3s or live lymphocytes (Figures 5B and D). The results revealed significant increases in the percentages of NKp46^+^ILC3s, along with increased MFI of NKp46 in NKp46^+^ILC3s, in jejunal LPLs on day 28 (Figures 5B and C). Intriguingly, the percentage of NKp46^–^ILC3s was reduced on day 28 (Figure 5D). These findings suggest that NKp46^−^ILC3s develop earlier than NKp46^+^ILC3s do in piglets.

To test our hypothesis that IFN-γ is produced primarily by NKp46^+^ILC3s, we performed further analysis to determine the production of IL-17A, IL-22, and IFN-γ from both NKp46^+^ILC3s and NKp46^−^ILC3s. The results revealed that both subsets could produce IL-17A, IL-22, and IFN-γ (Figures 5E–G). However, notably, NKp46^+^ILC3s secreted significantly greater amounts of IFN-γ than NKp46^−^ILC3s did (Figure 5G). These findings support this hypothesis and indicate that IFN-γ production is derived mainly from NKp46^+^ILC3s throughout the entire suckling period in piglets.

### Changes in jejunal ILC3s and IL-22 production after ETEC infection

**Figure 6:**
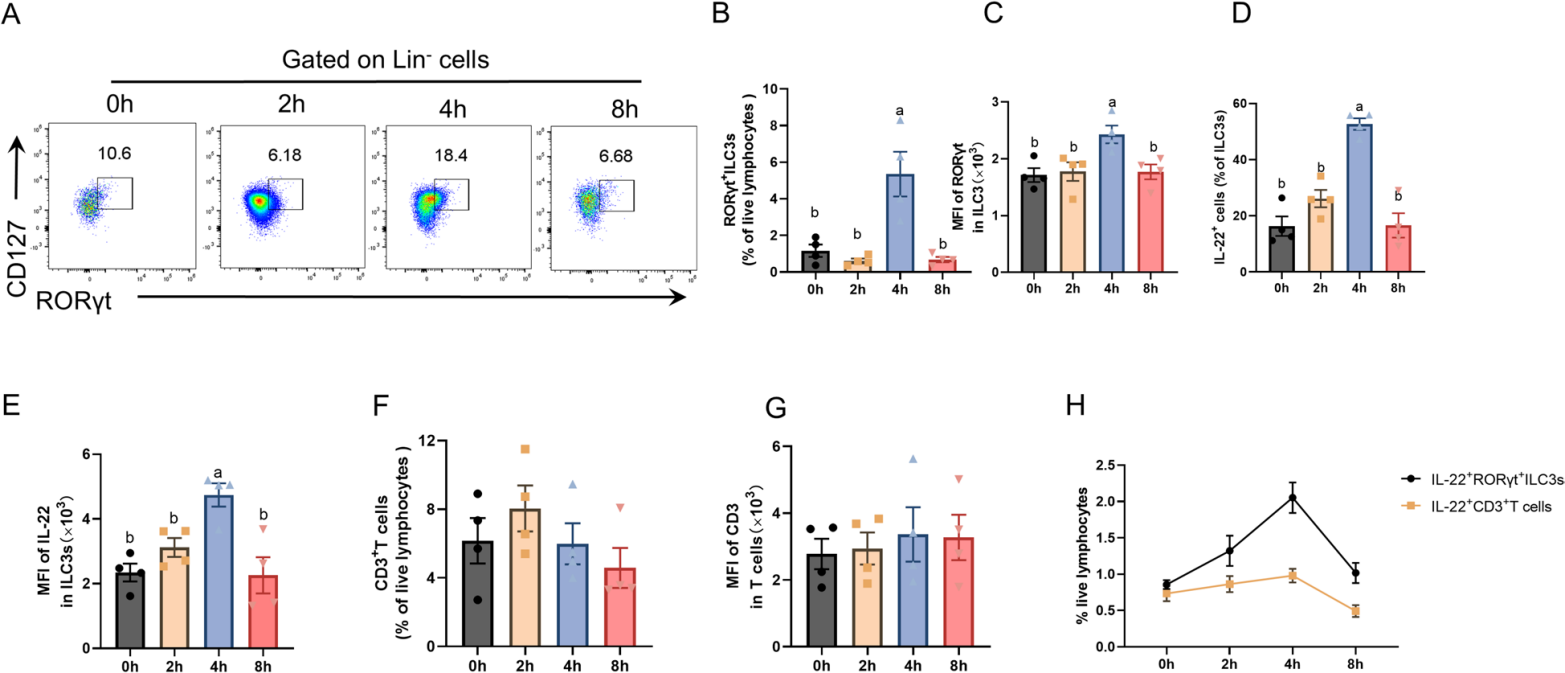
**Changes in jejunal ILC3s and IL-22 production after enterotoxigenic Escherichia coli infection.** Suckling piglets were challenged with enterotoxigenic *Escherichia coli* via oral gavage. The numbers in the flow plots represent the percentage of ILC3s at the lineage gates (**A**). Percentages of RORγt^+^ILC3s in live lymphocytes (**B**). MFI of RORγt in RORγt^+^ILC3s (**C**). Percentages of IL-22 in total ILC3s (**D**). MFI of IL-22 in ILC3s (**E**). Percentages of CD3^+^ T cells in live lymphocytes (**F**). MFI of CD3 in CD3^+^ T cells (**G**). Percentages of IL-22-producing ILC3s or CD3^+^ T cells in live lymphocytes (**H**). The data are presented as the means ± standard errors, n = 4. ^a–b^Different lowercase letters represent significant differences (*P* < 0.05).

To further explore the function of ILC3s in infection, this study constructed a model of suckling piglets with 10^11^ CFU of the ETEC strain by oral gavage. This work then isolated and analysed jejunal ILC3s after ETEC infection. Four hours after infection, the percentages of ILC3s among lineage-negative cells and live lymphocytes peaked (Figures 6A and B), and the MFI of RORγt in RORγt^+^ ILC3s also increased (Figure 6C). Moreover, the percentages of IL-22^+^ cells among total ILC3s were significantly increased (Figure 6D), and the MFI of IL-22 in ILC3s was greater at 4 h post infection (Figure 6E). However, the levels of IL-17A and IFN-γ produced by ILC3s did not significantly change after ETEC infection (data not shown). T cells are also a major source of IL-22 in mice and humans [35]. In the present study, the percentages and CD3 MFI of CD3^+^ T cells were not significantly different during ETEC infection (Figures 6F and G). In addition, compared with CD3^+^ T cells, RORγt^+^ILC3s produced significantly greater amounts of IL-22 (Figure 6H). These findings suggest that ILC3s play a significant role in the early response to ETEC infection by producing IL-22.

## Discussion

The period from birth to weaning is critical for immune system development in piglets [2]. During this time, the intestinal immune system gradually matures due to the colonization of the intestinal microbiota and the influence of environmental factors [36]. Innate immunity plays a crucial role prior to the development of adaptive immunity and regulates the response of the adaptive immune system [37–39]. Recent studies using single-cell RNA sequencing have identified ILC3s as the main type of innate lymphoid cells present in the jejunal lamina propria of pigs [17]. However, the development of jejunal ILC3s and IL-17A, IL-22, and IFN-γ remains largely unknown. The present study revealed the slow development of jejunal ILC3s in early life and a marked increase on day 28 after birth in piglets. Interestingly, IL-17A and IFN-γ secreted by ILC3s presented similar developmental patterns. These findings provide important insights into the regulation of intestinal ILC3 development and function, which can ultimately lead to improved disease resistance and reduced mortality in piglets.

There are many immune cells in the intestine, including T cells, B cells, myeloid cells, and ILCs. Among them, ILCs have been identified as CD45^+^ cells that lack lineage markers for T cells (CD3), B cells (CD19), and monocytes/macrophages (CD14) in humans [40, 41]. Studies in humans and mice have shown that ILC3s express the signature genes IL-7Rα (CD127) [42] and RORC (RORγt) [43], and this has also been confirmed in pigs [17, 44]. After CD45^+^Lin^−^CD127^+^ cells were gated, RORγt^+^ILC3s were identified in the present work. These findings demonstrate that the proportions of ILC3s within lymphocytes in the porcine intestine at a steady state are comparable to those observed in humans and mice, with ILC3s being the predominant subset of ILCs [32]. In addition, the present study revealed that the proportions of ILC3s in the jejunum of suckling piglets were significantly greater than those in the colon, which aligns with observations in mice and humans [5, 45]. Moreover, NKp46^−^ILC3s harbour precursors of NKp46^+^ILC3s and develop into NKp46^+^ILC3s [11, 34]. Specifically, the results of this study revealed that the proportion of NKp46^−^ILC3s among total ILC3s was slightly greater than that of NKp46^+^ILC3s in the early stages. The immune system of pigs is highly similar to that of humans [46], and pigs have been used extensively to investigate human nutrition and diseases [47–50]. These findings not only support the validity of the flow cytometry protocol used in the present study but also indicate that piglets can serve as a reliable model for studying ILC3s in the context of intestinal immunity.

In addition to ILC3s, NK cells, γδT cells, and T cells are the major immune cells that produce IL-17A, IL-22, and IFN-γ in humans and mice [23, 51–53]. To elucidate the cellular sources of these cytokines in the intestine of piglets, the present study employed flow cytometry to analyse the proportions of ILC3s, NK cells, γδT cells, and T cells. Throughout the entire observation period, the proportion of NK cells were the lowest, with no significant increasing trend. This finding also indicates that NK cells contribute the least to the production of IL-17A, IL-22, and IFN-γ in a steady state. Previous studies have shown that NK cells can be observed in foetal tissue during pig gestation; however, their functional capabilities are absent in the early stages [54]. Although γδT cells are the first T lymphocyte subset to appear early in life, the number of αβT cells gradually increases with increasing piglet age [54, 55], and CD4^+^ naive helper cells constitute the majority of the T-cell population after birth [56]. The findings of these works also indicated that the proportions of T cells were greater than those of NK cells and/or γδT cells throughout the suckling period. Moreover, T cells were the predominant sources of IL-17A, IL-22, and IFN-γ during preweaning, reaching the maximum level on day 21, which was consistent with the levels of these cytokines in the plasma and jejunum of piglets. Therefore, these cytokines may be produced mainly by T helper (Th) cells.

IL-17A, IL-22, and IFN-γ are key cytokines for ILC3 function [6, 18, 57]. This study examined these cytokines derived from ILC3s and revealed that the amount of IL-17A in ILC3s was consistent with that in jejunal ILC3s. IFN-γ-producing ILC3s remained steady in early life and then significantly increased on day 14, whereas there was no significant difference in IL-22 production by ILC3s during the suckling period. This difference may be explained by the different developmental patterns of ILC3 subsets [9, 11]. NKp46^+^ILC3s and NKp46^−^ILC3s produce different cytokines, resulting in different functions [9, 58]. In mice, NKp46^+^ILC3s produce high levels of IFN-γ, whereas NKp46^−^ILC3s produce lower levels after activation [9]. In the present study, the development of NKp46^+^ILC3s displayed consistent patterns with the changes observed in ILC3s. The percentages of NKp46^−^ILC3s significantly increased on day 14 and remained stable on day 21. Compared with those on days 1–7, they subsequently returned to an equivalent level on day 28. The developmental turning point occurred earlier than that of NKp46^+^ILC3s. The production of IFN-γ by NKp46^+^ILC3s in the jejunal lamina propria of suckling piglets was consistent with that of NKp46^+^ILC3s. This finding suggested that the marked increase in the proportion of jejunal ILC3s on day 28 was mainly the result of NKp46^+^ILC3 expansion.

ETEC is the most prevalent challenge causing illness and impairing gut health in nursing piglets, leading to a negative impact on the development of the pig industry and economic benefits [59]. Multiple studies have shown that ILC3s play important roles in pathogen infection and host homeostasis [60]. In the present study, higher levels of ILC3s and IL-22 were observed at 4 h postinfection than at 0 h, which coincided with the presence of watery diarrhea in the piglets. IL-22 is a key regulator of epithelial barrier function and repair [61]. Previous studies have demonstrated that NK cells produce IL-22, possibly providing protection to the gut epithelial barrier against ETEC infection [62, 63]. The present study revealed that the percentages of ILC3s were consistently greater than those of NK cells throughout the entire experiment. In addition, Th17 cells are also major producers of IL-22 [64]. This study further analysed IL-22 produced by CD3^+^ T cells and RORγt^+^ILC3s and revealed that IL-22 was derived mainly from RORγt^+^ILC3s rather than from CD3^+^ T cells. Research has shown that most IL-22^+^ cells lack CD3e expression in the ileum lamina propria of 7-week-old piglets on the basis of dual protein immunofluorescence/fluorescent RNA in situ hybridization [16]. However, whether IL-22 ^+^ cells express CD3 during ETEC infection needs to be further investigated.

## Supplementary Information

**Additional file 1**. **Flow cytometry strategy used to distinguish ILC3s and cytokines.** Histograms illustrating the gating strategy used for identifying ILC3sand their cytokines.

## Data Availability

All the data generated or analysed during this study are included in this published article.
