# Supplementary figures and images for: Developmental patterns of intestinal group 3 innate lymphoid cells in piglets and their response to enterotoxigenic Escherichia coli infection
Source: Vet Res. 2024 Dec 18;55:159. doi: 10.1186/s13567-024-01418-3 (PMC11654254; doi:10.1186/s13567-024-01418-3)

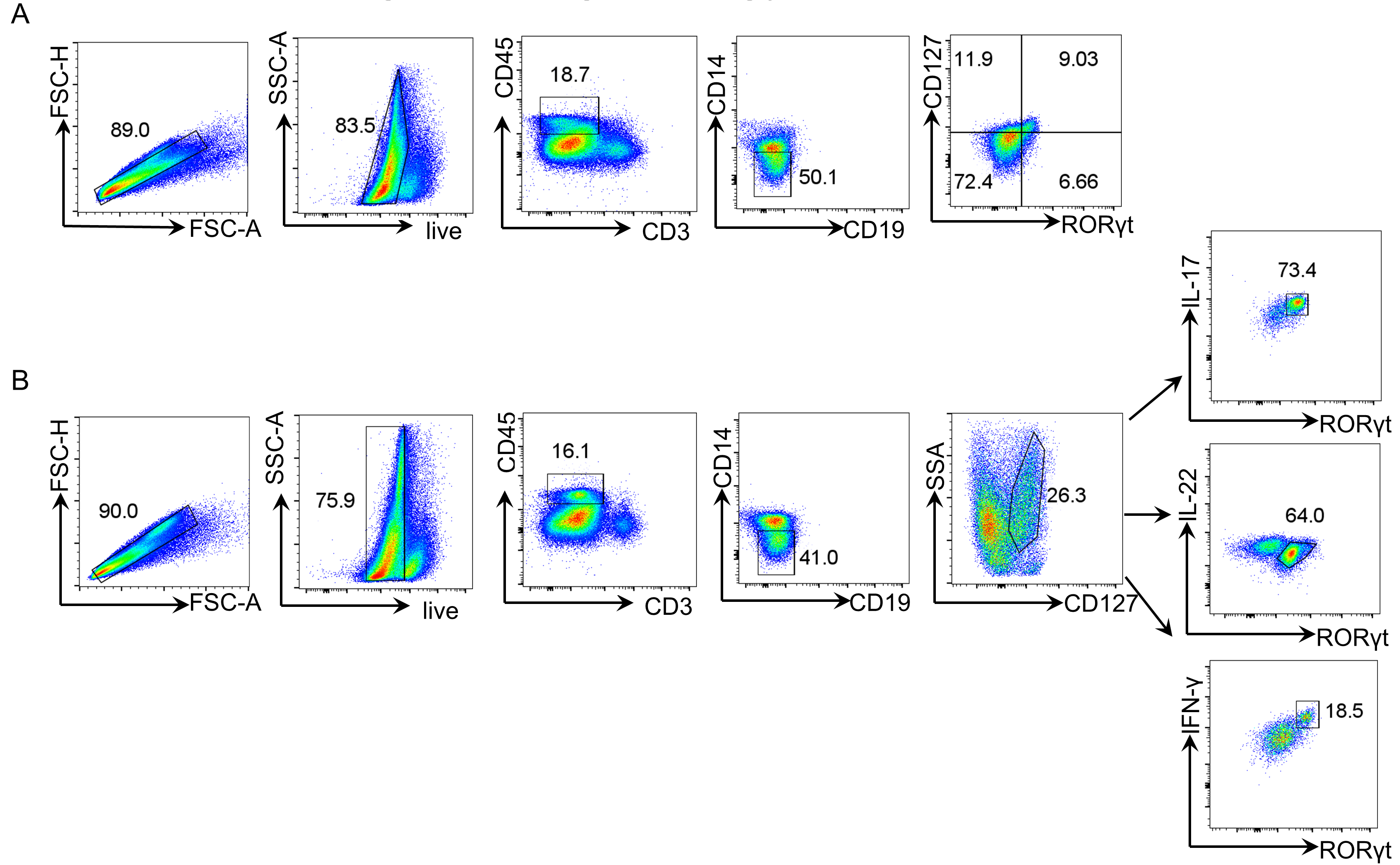

Supplement: Supplementary file 1 — Additional file 1. Flow cytometry strategy used to distinguish ILC3s and cytokines. Histograms illustrating the gating strategy used for identifying ILC3sand their cytokines. [file 13567_2024_1418_MOESM1_ESM.tif]
